# Exploring the longevity advantage of doctorates in Finland and Sweden: The role of smoking- and alcohol-related causes of death

**DOI:** 10.1177/1403494820969541

**Published:** 2020-11-12

**Authors:** Liina M. Junna, Lasse Tarkiainen, Olof Östergren, Domantas Jasilionis, Pekka Martikainen

**Affiliations:** 1 Population Research Unit, Faculty of Social Sciences, University of Helsinki, Finland; 2 The Max Planck Institute for Demographic Research, Germany; 3 Helsinki Institute of Urban and Regional Studies, University of Helsinki, Finland; 4 Department of Public Health Sciences, Stockholm University, Sweden; 5 Aging Research Centre (ARC), Karolinska Institutet, Sweden; 6 Demographic Research Centre, Vytautas Magnus University, Lithuania

**Keywords:** Alcohol, education, gender, mortality, smoking, social inequalities

## Abstract

Aims: Tobacco smoking and alcohol use contribute to differences in life expectancy between individuals with primary, secondary and tertiary education. Less is known about the contribution of these risk factors to differences at higher levels of education. We estimate the contribution of smoking and alcohol use to the life-expectancy differences between the doctorates and the other tertiary-educated groups in Finland and in Sweden. Methods: We used total population data from Finland and Sweden from 2011 to 2015 to calculate period life expectancies at 40 years of age. We present the results by sex and educational attainment, the latter categorised as doctorate or licentiate degrees, or other tertiary. We also present an age and cause of death decomposition to assess the contribution of deaths related to smoking and alcohol. Results: In Finland, deaths related to smoking and alcohol constituted 48.6% of the 2.1-year difference in life expectancy between men with doctorate degrees and the other tertiary-educated men, and 22.9% of the 2.1-year difference between women, respectively. In Sweden, these causes account for 22.2% of the 1.9-year difference among men, and 55.7% of the 1.6-year difference among women, which in the latter case is mainly due to smoking. **Conclusions: Individuals with doctorates tend to live longer than other tertiary-educated individuals. This difference can be partly attributed to alcohol consumption and smoking.**

## Introduction

Educational disparities in mortality are well documented [1]. However, most studies focus on the disadvantaged position of the lowest educated people, while the advantages of those with doctoral degrees over those with other tertiary education have received less attention [2,3]. Even though the proportion of the highly educated is growing, the processes behind life expectancy differentials at higher levels of education are unclear. Such evidence is also of value, as these vanguard groups may indicate frontiers of longevity eventually achievable for the entire population [4,5].

Alcohol use and smoking contribute to the overall educational health gap, but the magnitude of their contribution at higher levels of education [6] and in different settings is unknown. We examined to what extent the gap in life expectancy between doctorate and other educational groups is due to differences in smoking and alcohol use. Our research focused on Finland and Sweden – two Nordic countries with roughly comparable educational and welfare systems but differing levels of smoking- and alcohol-related mortality [7].

## Methods

We used register data for the total Finnish and Swedish populations linked with death records for 2011–2015 (Ethics Committee of Statistics Finland permission TK-53-1783-96; Central Ethical Review Board in Sweden permit Ö 25-2017). We excluded emigrants and those <40 years of age. By this age, education is well established.

Deaths and person-years were cross-tabulated by sex, 5-year age group and education. We calculated education-specific life expectancy at 40 years of age (e40) with 95% confidence intervals, using abridged life tables, with a final age band of ⩾85 years [8]. We then conducted an age and cause decomposition between those with doctorates and those with other tertiary education to estimate the contributions of smoking and alcohol consumption [9].

Education was measured as the highest completed degree: other tertiary (13+ years, International Standard Classification of Education (ISCED) 2011 level 5–7) and doctorate or licentiate (ISCED 8). In the Nordic countries, a licentiate follows a master’s degree and precedes a doctorate degree.

We defined alcohol-related deaths as alcohol-attributable diseases, mental-health conditions and poisoning (International Classification of Diseases 10th revision: F10, G312, G4051, G621, G721, I426, K292, K70, K852, K860, O354, P043, Q860 and X45) [10].

We estimated the mortality attributable to smoking using the indirect method developed by Preston et al. [11] in which age, year and sex-specific lung cancer death rates are used as indicators of exposure to smoking. The authors estimated a regression model for the association between deaths due to lung cancer and other causes for 20 countries. This model can then be applied to a population of interest to assess the share of all deaths attributable to smoking. The original coefficients were estimated for ages ⩾50 years. We used the extended estimates described in Martikainen et al., which also discusses the application of the method in social inequalities research [12].

## Results

In 2011–2015, 1.4% of Finnish men and 0.8% of women aged ⩾40 years held a doctorate or licentiate degree, while 28.0% of men and 32.0% of women were other tertiary educated (Supplemental Table SI). In Sweden 1.6% of men and 0.8% of women held a doctorate, and 27.3% and 31.3% were other tertiary educated, respectively. The share of those who were doctoral educated was small among the older age groups, particularly among women.

The doctorates had consistently higher life expectancies compared to the other tertiary educated (Table I). The gap was slightly wider in Finland than in Sweden, particularly among women. Smoking- and alcohol-related deaths contributed approximately 0.49 years to the 2.1-year difference in life expectancy among Finnish women – a smaller contribution when compared to almost a year among the Swedish women. The larger gap among Swedish women was mostly due to smoking.

**Table I.** Life expectancy at 40 years of age (e(40)), educational difference in e(40) and the contribution of smoking- or alcohol-related mortality to this difference among Finnish and Swedish men and women in 2011–2015.

	Education		Difference in e(40)	Contribution to difference in e(40), years		
	Tertiary e(40) (95% CI)	Doctorate e(40) (95% CI)		Smoking	Alcohol	Other
Finland						
Men	42.9 (42.8–43.0)	45.0 (44.5–45.5)	2.14 (100%)	0.69 (32.1%)	0.35 (16.5%)	1.10 (51.4%)
Women	47.3 (47.2–47.4)	49.4 (48.6–50.2)	2.10 (100%)	0.33 (15.5%)	0.16 (7.4%)	1.62 (77.1%)
Sweden						
Men	43.7 (43.6–43.7)	45.5 (45.2–45.9)	1.89 (100%)	0.36 (18.8%)	0.06 (3.4%)	1.47 (77.8%)
Women	47.2 (47.1–47.2)	48.8 (48.2–49.3)	1.61 (100%)	0.89 (55.1%)	0.01 (0.6%)	0.71 (44.3%)

CI: confidence interval; e(40): life expectancy at 40 years of age.

Among Finnish men, roughly half of the 2.14-year gap was attributable to smoking (32.1%) and alcohol (16.5%), whereas in Sweden these factors explained 22.2% of a similar gap, the majority of it due to smoking (18.8%).

## Discussion

We estimated life expectancy at 40 years of age for doctorates and licentiates and the other tertiary educated in Finland and Sweden, and decomposed the educational gap by cause of death. Those with doctorates had higher life expectancies compared to the other tertiary educated – a difference of a little over 2 years in Finland and slightly under 2 years in Sweden. These differences are substantial, although somewhat smaller than those between, for example, the tertiary and secondary educated (Supplemental Table SII).

Health-related behaviours such as smoking and alcohol use have previously been shown to contribute to the overall educational gap in mortality [6]. These factors also substantially contribute to differences in life expectancy between higher-educated groups. Our findings are generally in line with studies concerning the difference between basic and tertiary educated [13,14] or studies where income is addressed instead of education [12]. These results indicate that the social gradient in mortality at the very top of the hierarchy is at least partially due to similar factors that drive social differentials in mortality more generally. We found country differences in the expected direction: alcohol plays a larger role in Finland, while smoking is important among Swedish women [13,14].

Since we accounted for smoking- and alcohol-related behaviours, the remaining differences in life expectancy must be due to other factors. These include other health behaviours such as diet quality and exercise [15], and social and material advantages [16]. The more highly educated may also benefit first from new health-care innovations and improvements in contextual factors [17]. Furthermore, it is possible that some occupations requiring a doctoral degree are particularly beneficial to health, combining high status with the possibility of meaningful activity, even after retirement age [2].

The difference between the doctors and the other tertiary educated could also be due to direct health-related selection, as health-related problems may affect the probability of completing a doctoral programme. However, obtaining a doctorate is but one out of many challenging career paths available to university graduates, several of these more lucrative than a career in academia. It is therefore unclear whether health selection contributes to the high life expectancy of doctors – a fruitful topic for further study.

The role of the ongoing educational expansion – particularly among women – is also noteworthy. The Nordic countries have progressed far in this process, although they are not outliers within Europe where the proportions of the highly educated have increased to record highs, especially in the youngest cohorts [18]. Various individual characteristics such as socio-economic background and personality are associated with both education and mortality. As the share of the tertiary educated increases, this group will become more heterogeneous and should slowly lose some of their advantage related to direct and indirect health selection [1,19].

The strengths of this paper include the high-quality total population register-based data, and addressing the correlated behaviours of smoking and alcohol use. We were unable to include deaths in which alcohol was a contributory cause and deaths from external causes involving alcohol. Therefore, our estimate for alcohol may be conservative. While this underestimation is assumed proportional across educational groups, the true contribution of alcohol to educational differences in life expectancy is likely higher [10]. However, this does not affect the overall conclusion that alcohol substantially contributes to the gap in life expectancy between highly educated groups.

## Conclusions

In Finland and in Sweden, individuals with doctorates or licentiates had a longer life expectancy that those with other tertiary degrees. Benefits related to education show no signs of capping at university. Mortality due to smoking and alcohol use contributed to these differences, indicating that behavioural risk factors contribute to educational disparities across the full educational distribution. Moreover, these deaths are preventable. Thus, the results for this vanguard group highlight a potential for further gain in life expectancy, even among the tertiary educated.

## Supplemental Material

SJP969541_Supplementary_material – Supplemental material for Exploring the longevity advantage of doctorates in Finland and Sweden: The role of smoking- and alcohol-related causes of death Click here for additional data file. Supplemental material, SJP969541_Supplementary_material for Exploring the longevity advantage of doctorates in Finland and Sweden: The role of smoking- and alcohol-related causes of death by Liina M. Junna, Lasse Tarkiainen, Olof Östergren, Domantas Jasilionis and Pekka Martikainen in Scandinavian Journal of Public Health
