# Supplementary material for: Exploring the longevity advantage of doctorates in Finland and Sweden: The role of smoking- and alcohol-related causes of death
Source: Scand J Public Health. 2020 Nov 12;49(4):419–22. doi: 10.1177/1403494820969541 (PMC8135231; doi:10.1177/1403494820969541)
Supplement: SJP969541_Supplementary_material – Supplemental material for Exploring the longevity advantage of doctorates in Finland and Sweden: The role of smoking- and alcohol-related causes of death [file SJP969541_Supplementary_material.pdf]

Web Table 1. Age and cause of death decomposition of the difference in life expectancy between those with doctorate or licentiate degrees and the other tertiary educated [a] in Finland and Sweden in 2011-2015 by age group and sex. [b]

		Contribution of cause of death, in years			Overall contribution of age group, in years	Share of educational group within age group, %	
	Age group	Smoking	Alcohol	Other causes		Doctorates	Other tertiary
Finland, men	40	0.00	0.04	0.07	0.12	1.54	34.35
	45	0.00	-0.04	0.10	0.07	1.52	32.12
	50	-0.02	0.06	0.03	0.07	1.41	30.37
	55	0.03	0.09	0.14	0.25	1.41	28.99
	60	0.07	0.14	0.14	0.35	1.36	26.87
	65	0.09	0.05	0.24	0.39	1.45	26.86
	70	0.11	0.00	0.21	0.32	1.42	24.30
	75	0.05	0.02	0.14	0.20	1.09	20.19
	80	0.14	0.01	0.02	0.17	0.83	17.45
	85+	0.22	-0.01	0.00	0.21	0.72	16.34
	Total	0.69	0.35	1.10	2.14	1.38	28.03
Finland, women	40	0.00	0.01	0.04	0.06	1.49	51.36
	45	0.00	0.04	0.01	0.05	1.33	47.69
	50	0.00	0.02	0.06	0.09	1.21	43.13
	55	0.02	0.01	0.14	0.17	1.00	36.99
	60	0.01	0.04	0.15	0.20	0.78	29.56
	65	-0.02	0.03	0.07	0.08	0.64	25.83
	70	0.03	0.03	0.21	0.27	0.47	20.49
	75	0.06	-0.03	0.06	0.08	0.24	14.33
	80	-0.07	0.01	0.53	0.47	0.15	11.09
	85+	0.29	0.00	0.34	0.63	0.11	8.71
	Total	0.33	0.16	1.62	2.10	0.84	31.99
Sweden, men	40	0.00	0.00	0.08	0.07	1.70	31.59
	45	0.00	0.00	-0.01	-0.01	1.63	29.52
	50	0.01	0.00	0.11	0.12	1.65	28.99
	55	0.00	0.01	0.08	0.10	1.61	28.33
	60	0.04	0.02	0.10	0.15	1.66	24.75
	65	0.02	0.01	0.17	0.20	1.80	20.78
	70	0.03	0.01	0.15	0.19	1.60	17.81
	75	0.06	0.01	0.24	0.31	1.32	16.14
	80	0.07	0.00	0.30	0.37	0.90	14.06
	85+	0.14	0.00	0.24	0.38	1.70	31.59
	Total	0.36	0.06	1.47	1.89	1.64	27.25
Sweden, women	40	0.00	0.00	0.07	0.07	1.34	45.54
	45	0.01	0.00	0.02	0.03	1.13	40.19
	50	0.01	0.00	0.14	0.15	1.04	37.78

55	-0.01	0.00	0.10	0.09	0.93	36.19
60	0.06	0.00	0.06	0.12	0.76	33.25
65	0.08	0.01	0.13	0.22	0.66	28.44
70	0.03	-0.01	0.21	0.23	0.58	23.26
75	0.10	0.01	0.12	0.22	0.38	18.20
80	0.13	0.00	0.05	0.19	0.23	13.70
85+	0.48	0.00	-0.19	0.29	0.15	8.88
Total	0.89	0.01	0.71	1.61	0.81	31.34

[a] Education was defined as other tertiary education (13+ years. International Standard Classification of Education ISCED 2011 level 5-7). and doctorate or licentiate (ISCED 8). composition of the difference between two

[b] The decomposition method measures the overall contribution of each age group and cause of death to the total gap in life expectancy. calculated from age group –specific death rates and presented in years. A negative measure indicates a narrowing contribution to the gap. For example. smoking particularly contributed to the widening of the gap in older age groups among Finnish men and Swedish men and women. while the contributions were negligible or even offset in younger age groups. Meanwhile. alcohol most contributed around the age group 60-65 among all except for Swedish women. among whom the contribution of alcohol was very small (0.01 years or 0.6% of the gap).

Web Table 2. Life expectancy at age 40 (e(40)) with (e(40)) and without (e(40)n) the contribution of smoking or alcohol related mortality among Finnish and Swedish men and women in 2011-2015 by educational attainment. [a]

Country	Sex	Education	e40	e40n[b]	Difference e(40)n – e(40)
Finland	Men	Primary and lower secondary	36.9	42.9	6.0
		Upper secondary	39.5	43.8	4.3
		Tertiary	42.9	45.3	2.5
		Licentiate and doctorate	45.0	46.3	1.2
	Women	Primary and lower secondary	42.6	45.4	2.9
		Upper secondary	45.4	47.3	1.8
		Tertiary	47.3	48.4	1.1
		Licentiate and doctorate	49.4	50.0	0.6
Sweden	Men	Primary and lower secondary	38.9	41.1	2.2
		Upper secondary	41.1	42.9	1.9
		Tertiary	43.7	44.8	1.2
		Licentiate and doctorate	45.5	46.3	0.7
	Women	Primary and lower secondary	42.1	44.7	2.6
		Upper secondary	44.6	46.9	2.3
		Tertiary	47.2	48.6	1.5
		Licentiate and doctorate	48.8	49.0	0.3

Abbrevations: Life expectancy at age 40. e(40); Life expectancy at age 40 without the contribution of tobacco smoking and alcohol use. e(40)n

[a] Education was defined as: primary or lower secondary education (0-9 years. International Standard Classification of Education ISCED 2011 level 0-2). upper secondary education (10-12 years. ISCED 3-4). other tertiary education (13+ years. International Standard Classification of Education ISCED 2011 level 5-7). and doctorate or licentiate (ISCED 8).
[b] Without the contribution of deaths related to tobacco smoking and alcohol use.
